# Twenty years and still counting: including women as participants and studying sex and gender in biomedical research

**DOI:** 10.1186/s12905-015-0251-9

**Published:** 2015-10-26

**Authors:** Carolyn M. Mazure, Daniel P. Jones

**Affiliations:** Department of Psychiatry, Yale School of Medicine, New Haven, CT USA; Women’s Health Research at Yale, Yale School of Medicine, 135 College Street, Suite 220, New Haven, CT 06510 USA

**Keywords:** Women’s health research, Sex and gender, Women in clinical trials

## Abstract

**Background:**

This paper chronicles attempts in the United States over the past 20 years to fully represent women in clinical trials and ensure the study of sex and gender in biomedical research. We maintain that productive science with the aim of serving the public health requires examining the influence of sex and gender on health outcomes.

**Discussion:**

This section provides a historical perspective on the changes in recommendations and requirements of both the National Institutes of Health — the world’s largest single funder of biomedical research — and the U.S. Food and Drug Administration — the world’s most influential regulator of drugs and medical devices — for the acceptable conduct of research as it relates to sex and gender. We also cite all reports by the U.S. Institute of Medicine and the U.S. Congress’ General Accountability Office issued from 1990 to the present on the inclusion of sex and gender in research, and selected high-impact published studies that illustrate and document the paucity of, yet the need for, inclusion of females and consideration of sex and gender in research across an array of biomedical disciplines.

**Summary:**

The key message of this paper is that it has been 20 years since the first requirements to include women as well as men in clinical trials and analyze results by sex were mandated by a U.S. federal law, yet not nearly enough progress has been made. Recent signs of potential change in both policy and practice of scientific inquiry suggest much more progress may be within reach. However, awaiting a cultural shift to allow the study of sex and gender to be embraced is not seen as an effective strategy for change. Rather, specific instrumental recommendations are offered for how to include the study of sex and gender in research so as to increase our understanding and promotion of health for the benefit of all.

## Background

Now is the time to fully integrate the study of sex and gender into biomedical research — a goal that is essential to productive and reproducible scientific inquiry [1], and by so doing generate findings that advance the public health. We focus on efforts to reach this goal in the United States over the past 20 years since the 1994 implementation of a U.S. federal law (the 1993 NIH Revitalization Act) requiring researchers funded by the National Institutes of Health — the world’s largest single funder of biomedical research — to include women as well as men in clinical studies and analyze their results by sex or gender.

This report chronicles the changes in recommendations and requirements of both the National Institutes of Health and the U.S. Food and Drug Administration — the world’s most influential regulator of drugs and medical devices — for the acceptable conduct of research as it relates to sex and gender. All U.S. Institute of Medicine and General Accountability Office reports on this topic from 1990 to the present are cited, as are selected high-impact, published studies to highlight the paucity of, yet the continued need for, inclusion of females and consideration of sex and gender in research across an array of biomedical disciplines. These published studies are woven into the historical perspective to illustrate the impact of the legal milestones and policy initiatives. The scope of the report is from 1990 — the immediate prelude to the 1993 NIH Revitalization Act — to the present with an emphasis on recent developments through 2014. We do not attempt to review the status of women’s health research in the many other countries with scientists who are committed to and actively working on the advancement of this field.

The position advanced by this report is that steps have been taken in the United States to remedy the underrepresentation of women and the inadequate attention to sex and gender differences in research and regulatory approvals*.* However, progress has been painfully slow — stalling for long periods or sometimes reversing direction [2] — and, consequently, not nearly enough progress has been made.

For example, women remain underrepresented in clinical trials in cardiovascular disease, the primary killer of both women and men in the United States [3], and cancer, the nation’s second leading cause of death for both women and men [4]. Even when women have been included as subjects in clinical research, the influence of sex or gender is not widely analyzed and reported for various health outcomes [5]. Most laboratory studies continue to use only male animals and do not take note of how cells differ on the basis of sex, yet these very studies form the biological basis for human health studies and derivative treatments [6, 7].

Recognizing that the terms sex and gender are often used interchangeably, the Institute of Medicine’s (IOM) Committee on Understanding the Biology of Sex and Gender Differences provided a definition of each term in its 2011 report *Exploring the Biological Contributions to Human Health: Does Sex Matter?* We follow these definitions (see Table 1: Sex and Gender Definitions [8]) in our narrative but recognize, as did the IOM report, that current available definitions of these terms are not unambiguous and cannot always be used in a mutually exclusive fashion due to the interaction of biological and social elements affecting health outcomes [8].

**Table 1:** Definitions of sex and gender

The Institute of Medicine’s Committee on Understanding the Biology of Sex and Gender Differences provided these working definitions [8]	
Sex	“The classification of living things, generally as male or female according to their reproductive organs and functions assigned by chromosomal complement.”
Gender	“A person’s self-representation as male or female, or how that person is responded to by social institutions based on the individual’s gender presentation. Gender is rooted in biology and shaped by environment and experience.”

The current report concludes with recommendations regarding next steps in finally complying with the 20-year-old requirements set by the NIH, and the inclusion of sex and gender in biomedical research in a comprehensive and meaningful way. It is our view that further operational changes are needed to create a scientific environment in which sex and gender-sensitive approaches to research are embraced, and result in greater understanding of how women and men are differentially affected by health conditions. Importantly, we maintain that such change will result in findings for both women and men that can be translated into improved health and healthcare.

## Discussion

### Milestones in the move toward inclusion

#### NIH and FDA: initial investigations and responses

***1990:****U.S. General Accounting Office Report — Problems in Implementing Policy on Women in Study Populations*. The General Accounting Office (GAO; called the Government Accountability Office since 2004) is the investigative arm of the U.S. Congress and can provide reports on issues of concern to the nation. This GAO report concluded that the NIH “has made little progress in implementing its policy to encourage the inclusion of women in research study populations” and that the policy “has not been well communicated or understood within NIH or in the research community.” The report noted that the NIH announced the policy four years earlier, but did not publish policy guidelines until almost three years after the announcement. Furthermore, the NIH had done little to encourage researchers to account for sex or gender when analyzing study results [9].

***1990:****The NIH Office of Research on Women’s Health was formed* “in response to congressional, scientific and advocacy concerns that a lack of systemic and consistent inclusion of women in NIH-supported clinical research could result in clinical decisions being made about health care for women based solely on findings from studies of men — without any evidence that they were applicable to women.” Since its inception, the National Institutes of Health’s Office of Research on Women’s Health (ORWH) has promoted policies and provided funding for research on women’s health and on the influence of sex and gender on health, and the training of women’s health researchers within and beyond the NIH. A key part of the ORWH’s mission continues to be ensuring that women’s health is appropriately represented in NIH-supported research studies [10].

***1991:****NIH announced the start of the Women’s Health Initiative (WHI).* This set of studies using large samples of American women who had gone through menopause was begun to better understand treatment for cardiovascular disease, cancer, and osteoporosis — the major causes of death, disability and impairment in this population of women within the United States [11]. WHI studies collectively enrolled more than 161,000 women and included an observational study and three main clinical trials (on hormone therapy, dietary modification, and calcium/vitamin D supplementation). These studies represented the first nationally-representative clinical trials designed to study key health conditions affecting women. Hormone therapy was of particular interest as it was commonly prescribed and increasing in use. For example, between the mid-1960s and mid-1970s, “the number of postmenopausal estrogen prescriptions increased markedly from an estimated nine million to approximately 25 million” [12]. And population surveys in the 1970s indicated that of 30 million postmenopausal women in the United States, one-third were receiving estrogens, often to prevent heart disease [13]. Yet, despite this practice, only observational data were available to support the use of hormones for the prevention of cardiovascular disease.

***1992:****U.S. General Accounting Office Report — Women’s Health: FDA Needs to Ensure More Study of Gender Differences in Prescription Drug Testing.* This report highlighted that the use of prescription drugs was the most common form of medical treatment, and that available data showed sex differences in the metabolism of drugs as well as in drug interactions. Yet women were significantly underrepresented in the clinical trials used to test the efficacy for approximately 60 % of drugs surveyed. When women were included, data were not analyzed for most drugs to determine whether responses differed between women and men. The report concluded that the FDA “should ensure that the pharmaceutical industry consistently include sufficient numbers of women in drug testing to identify gender-related differences in drug response and that such sex differences are explored and studied” [14].

***1993:****The NIH Revitalization Act was signed into law on June 10, 1993.* For the first time, NIH-funded investigators were to be required to include women as well as men in human subject research. For clinical trials, the law required agency-funded investigators to “ensure that the trial is designed and carried out in a manner sufficient to provide for a valid analysis of whether the variables being studied in the trial affect women or members of minority groups, as the case may be, differently than other subjects in the trial” [15]. Cost was not allowed as an acceptable reason for exclusion of women and minorities. However, requiring the study of female animals and human tissues that cannot be linked to a living individual was not included in this legislation. This same Act established the NIH Office for Research on Women’s Health by law.

***1993:****U.S. Food and Drug Administration Guidance for Industry: Guideline for the Study and Evaluation of Gender Differences in the Clinical Evaluation of Drugs.* This guideline, published in July 1993, changed the FDA’s 1977 guideline excluding women of childbearing potential from participating in early phase drug studies, which “may have led to a more general lack of participation of women in drug development studies, and thus to a paucity of information about the effects of drugs in women” [16]. The new guidance allowed, but did not require, representative inclusion of women in phase 1, 2 and 3 trials, and endorsed, but did not require, analysis of data on sex differences because the FDA did not “perceive a regulatory basis for requiring routinely that women in general … be included in particular trials.” The agency did encourage consideration of the effects of the menstrual cycle and of exogenous hormone therapy on drug treatment outcomes, and the effect of drugs on oral contraceptives’ efficacy, when feasible (see Table 2: FDA Clinical Trials for New Drug Approvals [17]).

**Table 2:** FDA clinical study phases for new drug applications

“The new drug application is the vehicle through which drug sponsors formally propose that the FDA approve a new pharmaceutical for sale in the United States” [17]	
Phase 1	Emphasizes the assessment of safety - how a new drug is metabolized and excreted, how a drug should be given, how often, at what dose. It is usually conducted with a small number of healthy volunteers.
Phase 2	Continues to test safety and begins to evaluate how well a drug works by comparing it with a different treatment, usually a placebo or another drug. Conducted in a larger sample.
Phase 3	Tests the efficacy of a new drug in comparison to the current standard, evaluates dosages and overall risk-benefit of the drug. Conducted in a large numbers of volunteers.
Phase 4	Studies post-approval use of a drug if the FDA judges the need to gather population data on drug safety.

***1994:****Requirements of the 1993 NIH Revitalization Act took effect.* In implementing the 1993 law, the *NIH Guidelines on the Inclusion of Women and Minorities as Subjects in Clinical Research* narrowed the requirement of including women in clinical trials to phase 3 trials only (see Table 3: NIH Clinical Trials for Human Subject Research [18]). However, these guidelines stated that such NIH-funded investigations must ensure that sufficient numbers of women are included in a phase 3 clinical trial “such that valid analyses of sex differences in the effects of interventions can be accomplished” [19].

**Table 3:** NIH clinical trial phases for human subject research

“Biomedical clinical trials of an experimental drug, treatment, device, or behavioral intervention may proceed through four phases” [18]	
Phase 1	Tests a new intervention in a small group of human subjects for the first time to evaluate efficacy and safety.
Phase 2	Tests the efficacy of a new intervention in a larger group (usually several hundred) to further evaluate efficacy and safety.
Phase 3	Compares a new intervention to standard or to other experimental interventions in large groups (several hundred to thousands) to determine efficacy and to monitor and collect data on adverse effects.
Phase 4	Assesses after-market effectiveness in the general population and collects data on any adverse effects associated with widespread use.

***1998:****U.S. Food and Drug Administration Final Rule: Investigational New Drug Applications and New Drug Applications.* These new regulations published in the *U.S. Federal Register* defined new drug application requirements pertaining to effectiveness and safety data for demographic subgroups, specifically gender, age and racial subgroups. The rules did not impose any new mandates on the conduct of studies for new drug applications, requiring only safety and efficacy data that already had been collected be presented separately for these demographic subgroups, including men and women. The FDA did not require any discussion or analysis of these data [20].

***2000:****U.S. General Accounting Office Report — Women’s Health: NIH Has Increased Its Efforts to Include Women in Research.* This May 2000 report concluded that significant progress was being made in increasing representation of women in clinical trials, spurred by the fact the inclusion of subgroup populations had become a matter of scientific merit when research grants were assessed for funding. However, the report found that studies still were not routinely being designed to enable analyses by sex, and when researchers were analyzing outcomes by sex, these results were not always published [21].

***2000:****Building Interdisciplinary Research Careers in Women’s Health (BIRCWH), a mentored career-development program, began to train junior faculty in interdisciplinary research on women’s health and sex differences.* This continuing program is a collaborative effort to ensure that the influence of sex and gender on health is studied by the next generation of scientists. It is funded by the NIH Office for Research on Women’s Health and its Institute partners. Since it began, the ORWH and its BIRCWH program co-sponsors have awarded 77 training grants to 39 institutions supporting more than 542 junior faculty [22].

***2000****: FDA Amendment for Investigational New Drug Applications addresses products intended for life-threatening conditions that affect both genders.* In June 2000, the FDA issued a new regulation allowing the agency to halt research on drugs for life-threatening diseases and conditions if men or women who have the condition are excluded from study based on a perceived risk to their reproductive potential. The rule does not apply to conditions relevant to only one sex or gender, and it does not require researchers “to enroll or recruit a specific number of men or women with reproductive potential” rather it seeks to remedy the historic exclusion of women with a potentially deadly condition [23].

***2001:****U.S. General Accounting Office Report — Drug Safety: Most Drugs Withdrawn in Recent Years Had Greater Health Risks for Women.* This January 2001 GAO report concluded that, of the 10 prescription drugs approved by the FDA and subsequently withdrawn from the U.S. market since 1997, eight of these drugs constituted a greater risk to the health of women than men [24]. In a subsequent 2007 review, it was again demonstrated that women suffered more adverse effects from prescription drugs than men [25].

***2001:****U.S. General Accounting Office Report — Women Sufficiently Represented in New Drug Testing, but FDA Oversight Needs Improvement*. This July 2001 GAO report concluded that approximately one-third of applications to the FDA for new drugs failed to meet the agency’s 1998 regulation change requiring presentation of safety and efficacy data by sex if data were already collected. In addition, the GAO found that the FDA had not yet exercised its power to suspend research on life-threatening conditions if men or women were excluded based on their potential to reproduce, though the GAO report did not examine if such a sanction should have been used in any particular instance. And while the GAO determined that women accounted for 52 % of study subjects for new drug applications, women were only 22 % of those participating in the small first phase trials used to determine dosing for later, larger efficacy trials. The report concluded that the FDA had “not effectively overseen the presentation and analysis of data related to sex differences in drug development,” noting as an example that FDA reviewers failed to comment on applicants’ failures to include dosing adjustments based on sex even though one-third of applications “specified that the concentrations of the drug in the bloodstream were greater in people who weighed less, such as women.” The GAO recommended that the FDA employ management tools to enforce its regulations addressing sex and gender data in drug testing [2].

***2001:****Institute of Medicine Report — Exploring the Biological Contributions to Human Health — Does Sex Matter?* This IOM report concluded that the study of sex and gender differences is “evolving into a mature science.” It provided recommendations for “establishing the conditions” to encourage this science, and eliminating “barriers to its advancement.” The report emphasized that, “Being male or female is an important basic human variable that affects health and illness throughout the life span,” and recommended that “sex should be considered when designing and analyzing studies in all areas and at all levels of biomedical and health-related research” [8].

#### Emergence of research illustrating the importance of studying women

***2002 and 2004****: Two Women’s Health Initiative trials are halted*. The most prominent of the Women’s Health Initiative trials were two hormone therapy trials designed to test the effects of the most commonly prescribed estrogen and combined estrogen/progesterone therapy at the time of initiation of the trials. In particular, the effects of hormone therapy on preventing cardiovascular disease, fractures, and breast and colorectal cancer were studied [26]. Both parts of these hormone therapy trials were halted early, in 2002 and 2004, respectively, because investigators determined that estrogen did not appear to be protective against cardiovascular disease for which it was generally prescribed and that health risks outweighed benefits [27]. Despite subsequent controversy about the value versus adverse effects of estrogen use, this was the first attempt to investigate the benefits and risks of this widely prescribed hormone in a large nationally-representative longitudinal sample of women.

***2005:****The first randomized trial of low-dose aspirin for cardiovascular disease prevention focusing on women was published*. In 1989, the first U.S. randomized trial of aspirin for cardiovascular disease prevention was published, having enrolled 22,071 men and no women [28]. Part of the Physician’s Health Study supported by the National Heart, Lung and Blood Institute and published in *The New England Journal of Medicine*, this male-only study showed that aspirin taken every other day substantially reduced risk of a first heart attack in men 50 years of age and older, and might increase stroke risk. The findings in this study left women and their care providers uninformed as to whether this preventive strategy would help, harm or have no effect on women. The answer for women came in 2005 in a study also published in *The New England Journal of Medicine*. This Women’s Health Study of nearly 40,000 women, 45 years of age or older who were monitored for 10 years, showed that aspirin every other day lowered the risk of stroke significantly for women 65 years and older without reducing coronary artery disease risk in women under 65 — clearly different effects than in men [29]. Aspirin provided the same primary preventive benefit in women older than 65 as it did in men 50 years and over.

***2005:****American Academy of Orthopaedic Surgeons (AAOS)/National Institutes of Health Workshop summary and review of studies on gender differences in orthopedic medicine.* This publication in the *Journal of Bone and Joint Surgery* detailed an April 22–25, 2004 workshop sponsored by the AAOS, the leading provider of musculoskeletal education to orthopaedic surgeons, and the NIH. The workshop was convened to complement the Institute of Medicine’s 2001 report: *Exploring the Biological Contributions to Human Health: Does Sex Matter?* As reported from this workshop, “Musculoskeletal problems, such as osteoarthritis, osteoporosis, spinal disorders, and fractures, comprise an extremely large proportion of visits to primary care and orthopaedic physicians; all have a higher prevalence in women. Yet most clinicians are unaware that the sexual differences associated with these problems are the result of inherent differences in biology at the cellular and molecular level.” [30].

***2008:****Change in life expectancy and mortality trends in U.S. counties.* In a finding specific to the United States compared to Western Europe, Australia, New Zealand and Japan, life expectancy was found to be falling for a significant number of American women for the first time since the influenza epidemic of 1918 [31]. The study showing this, published in *PLoS Medicine* [32], used federal mortality statistics and U.S. Census population data to estimate sex-specific life expectancy for U.S. counties for every year between 1961 and 1999. From the early 1980s, life expectancy significantly decreased in 11 U.S. counties for men and in 180 counties for women (the majority of which were in poor areas and in the southern United States). Smoking, obesity and high blood pressure largely led to the higher rates of death for women.

***2009:****Review of sex-related influences on pain.* As concluded in this review in *The Journal of Pain*, recent findings demonstrate that women have a greater risk for many pain syndromes than do men, report greater pain after invasive procedures, and greater sensitivity to most forms of experimentally induced pain. The authors recommended ways to promote progress in pain research stating that “both preclinical and human studies should routinely include subjects of both sexes.” As reported, “The NIH requires this for human studies; however, nonhuman pain research continues to eschew females. Given that the clinical pain conditions to which preclinical research is intended to apply are female-predominant, one could argue that preclinical research that excludes females is incomplete at best or invalid at worst” [33].

***2009:****Studies on the relationship of the sex of patients to the influence of general anesthesia.* In reviewing physiological and pharmacological differences between women and men relevant to general anesthesia and recovery after surgery, findings published in *Anaesthesia and Intensive Care* indicated that women awaken from anesthesia faster than men, suggesting less sensitivity to the hypnotic effects of anesthesia, but have slower recovery from anesthetic drugs due to higher rates of complications or adverse effects from general anesthesia [34]. Two years after publishing this review, the authors then confirmed their findings using a prospective, matched cohort design with 500 patients (247 women and 253 men) who received a general anesthetic for elective surgery. This study in the *British Journal of Anaesthesia* showed women to be less sensitive to the drugs used, to wake faster and to have poorer recovery than men. These outcomes appeared to be due to sex differences in the pharmacodynamic effects of the anesthetic drugs. Subgroup differences were also noted in that premenopausal women compared to postmenopausal women had worse recovery [35].

#### Further reports and studies, and a new federal law

***2010:****The journals Nature and Science published articles on the paucity of female animal models in basic research.* Each of these prominent journals acknowledged the very important lack of information in many areas of health due to the absence of female subjects in studies using model systems [36, 37]. Scientists discussed the problem at a March 8–9, 2010 workshop convened in San Francisco by the Institute of Medicine’s Forum on Neuroscience and Nervous System Disorders [38]. “In a number of disciplines, researchers simply don’t study females, and there is so much evidence for sex differences at all levels of biological organization that to only study males, and assume the results apply to females, is just wrong” [36].

***2010:****Institute of Medicine Report — Women’s Health Research: Progress, Pitfalls and Promise.* This 2010 report assessed progress in addressing health conditions that are key to women’s health. The focus was on a number of diseases or syndromes that are more common or serious for women, or have different treatments for women than men, or for which there was a clear unmet need for research regarding women. The report found major progress in breast cancer, cardiovascular disease (CVD) and cervical cancer. The report credited the translation of new research findings in the progress made in treating these illnesses. In addition, consumer demand for results were cited in the case of breast cancer, and behavioral changes such as decreased smoking in the case of CVD. In the case of cervical cancer, research-based improvements in diagnosis and screening were credited as well as the development of a vaccine to prevent human papillomavirus (HPV), the cause of most cervical cancer [39].

The report found that research has contributed to some improvements for women in treating depression, HIV/AIDS and osteoporosis. Unfortunately, the report found little research-led progress for other conditions they examined including unintended pregnancy, maternal morbidity and mortality, autoimmune diseases, alcohol and drug addiction, lung cancer, gynecological cancers other than cervical cancer, non-malignant gynecological disorders, and Alzheimer’s disease. Also of note, the report found “inadequate enforcement of requirements that representative numbers of women be included in clinical trials,” and inadequate reporting of results on women thus impeding “identification of potentially important sex differences [and slowing] progress in women’s health research and its translation to clinical practice” [39].

***2010:****NIH Office of Research on Women’s Health — Moving into the Future with New Dimensions and Strategies: A Vision for 2020 for Women’s Health Research*. This report provided a comprehensive summary of “a two-year strategic planning process involving more than 1500 leading scientists, public policy experts, women’s health advocates, health care providers, elected officials and the public in five U.S. regional scientific meetings” [10]. In generating research priorities, the plan emphasized that it “can benefit both women and men by increasing our understanding of the role of sex/gender factors in differential disease risk, vulnerability, progression and outcome.” The report also predicted that “over the next decade, the exploration of biological sex differences at multiple levels, from genes to hormones to complex systems, will be greatly accelerated.” Yet, “research will fall far short of its promise to usher in an era of personalized medicine unless the contribution of biological sex to the diversity of health outcomes is better understood and this knowledge applied in the development of the next generation of interventions and medical technologies” [10].

***2010:****Review of women, depression and treatment development.* This review of various pharmacological, behavioral and other interventional clinical trials, published in the *Journal of Women’s Health*, examined the inclusion of women as study participants and the use of gender-specific analyses. This study found that, of 150 randomized clinical trials published in 2007, with women averaging 56 % of the enrolled volunteers, half the studies did not analyze results by gender. Of 768 ongoing clinical trials, 89 % reported recruiting both women and men, but investigators reported an intention to analyze the results by gender in less than 1 % of these studies [40]. Yet, the World Health Organization, which ranked depression as the leading cause of disability worldwide as of October 2012, indicated that depression affects more women than men [41].

***2011:****Review of sex and gender differences in cardiovascular disease prevention.* Published in *Circulation,* this review reiterated once again that more women than men die each year in the United States from cardiovascular disease — the greatest cause of mortality for both women and men. It pointed out that inclusion of women in clinical trials for cardiovascular disease remained controversial as some examinations of the data suggest an increase in the proportion of women while others do not. Nonetheless, parity in inclusion had not been achieved and analysis of outcomes by sex remained minimal with evidence of such analyses in about one-third of studies [42].

***2011:****National Research Council Report — Explaining Divergent Levels of Longevity in High-Income Countries.* This report concluded that over the past 25 years “the United States has been falling steadily in the world rankings for life expectancy, a surprising development given the U.S. spends more on health care than any other nation.” Moreover, the reduction in the rate of increased life expectancy in the United States compared to other countries examined “is starker for women than for men.” Smoking was named a primary risk factor for reducing life expectancy in women, followed by “obesity, diet, exercise and economic inequality” [43].

***2011:****Review of studies of emergency medicine for inclusion of gender effects on health outcomes*. This review found that the majority of published studies which focused on emergency medicine from January 2006 to April 2009 reported gender as a demographic variable in 79 % of 750 studies. Yet, only 18 % of the studies examined health outcomes by gender. As pointed out in this study, published in the journal *Academic Emergency Medicine*, there is a tremendous need for gender-specific analyses in this field because, “Emergency physicians as front-line clinical specialists can directly advance patient care by understanding how gender-specific approaches may affect evaluation and management of diseases in the acute setting” [44].

***2011:****Institute of Medicine Report — Sex Differences and Implications for Translational Neuroscience Research.* Presentations and discussions from an IOM workshop held in San Francisco March 8–9, 2010 focused on why it was important to study sex differences in neuroscience and the need to translate findings to advance the public health. The content for discussion focused primarily on four disorders or conditions with clear gender differences or greater health burdens for women: pain, depression, sleep, and multiple sclerosis/neuroinflammation. The report indicated that, “sex difference research offers the opportunity to determine why one sex may be more predisposed to certain diseases, or have worse outcomes, while the other sex is protected. Therefore, the results from sex differences research will have a significant impact on the public health of both sexes” [38]. The key roles of scientific journals in reporting sex differences, and of industry in ensuring that sex differences are evaluated in drug development were highlighted.

Among the barriers to advancing our understanding of neurological disorders, the report included the lack of attention to the sex of cells because “every cell has a sex and sexual genotype (i.e., XX for females and XY for males) which can affect the pathophysiology and prevalence of some diseases,” the predominant use of male animal models in research, inadequate and inconsistent analysis and reporting of study results by sex, and reluctance of industry to conduct clinical trials that allow analysis by sex [38].

***2012:****Review of studies for FDA premarket approval of cardiovascular devices.* A critique of the FDA’s actions regarding the development of sex-specific data for medical devices, particularly cardiovascular devices, indicated that women were not well represented in clinical trials for these devices, and safety and efficacy by sex were not being adequately examined [45]. For example, a review of 78 high-risk cardiovascular devices receiving premarket approval from the FDA between 2000 and 2007, published in *Circulation: Cardiovascular Quality and Outcomes* [46], showed that the FDA evidence summaries did not report sex of participants in 28 % of 123 studies, and studies reporting enrollment by sex had an average of 67 % men with no indication of increased inclusion of women over time.

Forty-one percent of the studies had “a gender bias comment or analysis,” which could mean that women were not appropriately represented or that when women were studied, a sex difference was found (see Table 4: FDA Directive Requiring “Gender Bias” Analyses for Medical Devices [47]). In fact, a quarter of these analyses (12 of 47) found differences by sex either in safety or effectiveness. Such “gender bias” comments or lack of sex-specific analyses would run counter to the 1994 FDA directive [47] stating that “gender bias” should be reduced by studying an appropriate ratio of male and female subjects as well as providing safety and effectiveness data by gender. The FDA also had convened workshops in 2008 to inform guidance on this matter and issued draft guidance in 2011 with recommendations for increasing the number of women and conducting sex-specific analyses.

**Table 4:** FDA’s directive requiring “gender bias” assessment for medical devices

The FDA’s Office of Device Evaluation directed industry, when applying for approval of a new medical device, to address “gender bias” from two aspects in all Premarket Approval Applications and Summaries of Safety and Effectiveness Data by responding to the following questions [47]
•“Was the selection ratio of men versus women in the study reflective of the underlying distribution of the disease for that given age group, ethnic group, stage of disease, etc.? Was any selection bias on the basis of gender identified during review?”
•“Was there any difference in the safety and effectiveness of the device based on gender? For example, was the device more/less effective in women?”

***2012:****U.S. Congress enacted the 2012 Food and Drug Administration Safety and Innovation Act, signed into law July 9, 2012.* Section 907 of the law directed the FDA to review the inclusion and analysis of demographic subgroups in applications for drugs, biologics and devices by sex, race and ethnicity, and age [48]. The review was to take the form of a progress report of current practices and planning for further improvement, and a subsequent Action Plan with recommendations for enhanced enrollment and analysis of subgroups, and communication of data to health care professionals and the public.

***2013:****FDA released a progress report on the Collection, Analysis, and Availability of Demographic Subgroup Data for FDA-Approved Medical Products.* In August 2013, the FDA reported the results of an agency-wide working group’s investigation of 72 product applications approved in 2011 and offered areas of improvement that would form the basis of the following year’s Action Plan. The progress report “concludes that the statutes, regulations, and policies currently in place generally give product sponsors a solid framework for providing data in their applications on the inclusion and analysis of demographic subgroups.” The report also states that, “In general, sponsors are describing the demographic profiles of their clinical trial participants, and the majority of applications submitted to FDA include demographic subset analyses” [49]. This suggests that although the FDA supplies the necessary information on how sponsors should provide subgroup data and analysis, there is not consistent reporting by sponsors.

***2013:****Review of studies to determine inclusion of minorities and women in clinical trials on cancer.* Published in *Cancer,* this study examined 304 scientific publications reviewing both the diversity of and reporting on study participants in 277 treatment trials and 27 prevention trials conducted in the decade between 2001 and 2010. The large majority of subjects (greater than 80 %) were white and nearly 60 % were male. These data also were compared to an earlier study examining inclusion of women and minorities from 1990 to 2000 with the conclusion that, “Women and racial/ethnic minorities remain severely underrepresented in cancer clinical trials, thus limiting the generalizability of cancer clinical research” [4].

#### Current climate: change is possible

***2014:****CBS’s “60 Minutes” aired “Sex Matters: Drugs Can Affect Sexes Differently” on February 9, 2014.* With a segment by Lesley Stahl, this news show vaulted the issue of underrepresentation of women in clinical trials and lack of attention to sex and gender differences in research to national prominence, highlighting that, “More and more, scientists are realizing that the differences between the sexes are dangerously understudied.” The segment focused on the example of the FDA cutting the dose recommendation for Ambien in half for women because women metabolize the sleep-aid drug differently than men, leaving more of it in their system the next morning and putting them at greater risk of accidents due to impaired functioning. The difference was noted in a study analysis years earlier but nothing was done about it before the drug went to market. Although not as widely prescribed as Ambien, several other drugs have been identified by the FDA that require sex-sensitive prescribing [50].

***2014:****U.S. Surgeon General’s Report — The Health Consequences of Smoking: 50 Years of Progress.* Marking the 50th year since the 1964 U.S. Surgeon General’s report warned of the health hazards of smoking, this 2014 report stated that the lifetime quit ratios for “ever smokers,” meaning the percentage of those who had ever smoked a cigarette and then stopped smoking, was almost the same for women and men [51]. However, in any given quit attempt, women are less successful than men, indicating that women have more difficulty quitting [52]. Women are also more likely than men to relapse to smoking after quitting [53]. Moreover, female smokers may be at higher risk for lung cancer [54], earlier age of onset for breast cancer [55], and, compared to nonsmokers, have a 25 % higher risk than men for coronary heart disease [56].

***2014:****Letter to the FDA from U.S. Senators on April 30, 2014.* A bipartisan group of U.S. Senators sent a letter to the FDA requesting that the agency include in its forthcoming Action Plan a requirement for proportional representation of women and minorities in clinical research, assurance that analyses of subgroups are conducted, and a process to ensure the progress of the plan is tracked and results are made available to the public. They voiced their shared concern “about recent evidence that women still are not being adequately represented in clinical trials. As a result, medical treatments may not be as safe and effective as they may expect” [57]. One month later, a bipartisan group of members of the U.S. House of Representatives endorsed and delivered the same letter to the FDA [58].

***2014****: Letter to the Comptroller General from members of Congress on April 30, 2014.* In a letter to the Comptroller General, 10 Democratic U.S. Senators and members of the House of Representatives noted that 20 years had gone by since passage of the NIH Revitalization Act requiring appropriate representation of women and minorities and analysis by sex for NIH-funded studies. Given that it had been 15 years since the Government Accountability Office updated progress on these requirements, the members of Congress called on the GAO in this letter to the U.S. Comptroller General to investigate and issue a report on the participation of women in clinical trials broken down by disease and phase of the trial. They also asked the GAO to report the percentage of research on conditions affecting mostly women as well as both women and men, the ability of researchers to analyze data by sex and gender based on available sample sizes and their commitment to doing so, the factors affecting the enrollment of women in clinical trials, and the current oversight of policies for reporting on these issues by the National Institutes of Health [59]. As of October 5, 2015, these congressional leaders were awaiting the report.

***2014:****Review of studies for sex bias in surgical research.* This review in *Surgery* was the first to examine sex bias in the 618 basic and translational science studies published from 2011 to 2012 in prominent surgery journals. They found that when studying animals, researchers did not report on the sex 22 % of the time, and among those who did report the sex, 80 % used only males. Among the cellular studies, 76 % did not report the sex of the cell, and among the 24 % who did specify sex, 71 % used only male cells. “For publications on female-prevalent disorders, such as thyroid and cardiovascular disease, in which one would expect a larger number of publications studying females, only 12 % studied females or both sexes” [60]. The authors also found that, over time, sex disparity in surgical research had worsened, in that male-only studies were increasingly published despite the fact that “women manifest, progress, and react differently than men for many disease processes, including but not limited to cardiovascular disease, lung cancer, depression, obesity, osteoporosis, thyroid disorders, multiple sclerosis, and Alzheimer’s disease” [60].

***2014:****National Institutes of Health on May 15, 2014 announced a change in research policy, calling for balancing of sex in cell and animal studies.* Increasing the participation of women in NIH-funded clinical research in the two decades since the 1993 NIH Revitalization Act has helped expand knowledge about the effects of sex and gender on health outcomes. However, according to both the current Directors of the Office of Research on Women’s Health and the NIH, “There has not been a corresponding revolution in experimental design and analyses in cell and animal research — despite multiple calls to action.” They point out that “inadequate inclusion of female cells and animals in experiments and inadequate analysis of data by sex may well contribute to the troubling rise of irreproducibility in preclinical biomedical research” and that “the NIH is now actively working” to confront this serious issue by introducing improved policies in phases beginning in October 2014 [7].

***2014:****The Society for Women’s Health Research supports the NIH policy change on preclinical research.* Founded in 1990 to advocate for greater public and private funding for research on women’s health and gender differences, the society endorsed proposed changes to require inclusion of animals and cell types of both sexes and stated that, “Requiring sex-based information in preclinical studies as well as sex and gender-specific information in all phases of clinical research is expected to prevent life-threatening medical errors and unnecessary health risks for women” [61]. The Society continues to be highly active in education, advocacy and the promotion of health research examining differences between women and men.

***2014:****FDA announced its Action Plan to Enhance the Collection and Availability of Demographic Subgroup Data.* Section 907 of the FDA Safety and Innovation Act of 2012 required the FDA to issue an Action Plan on the inclusion and reporting of demographic data on subgroups including women and minorities. In April 2014, the FDA convened a public hearing to provide an update on their planning and gather comments [62]. Issued in August 2014, the Action Plan detailed 27 specific future actions that the FDA indicated it will take in order to increase the enrollment and participation of subgroups in clinical drug trials, and improve the quality and transparency of demographic subgroup data — which then can be applied to recommendations for the use of medical products [63].

***2014:****Federal agencies collaborating on women’s health research.* The NIH Office of Research on Women’s Health (established 1990) and the FDA Office of Women’s Health (established 1994) plan to collaborate on a national campaign to promote the importance of clinical trial participation focusing on women [63]. These two offices have a history of partnering to support research on women’s health and sex differences, for example, in funding “Specialized Centers of Research on Sex and Gender Factors Affecting Women’s Health” in various academic health centers across the nation [7]. They also have jointly developed and offered, since 2006, an online series of courses designed to promote research in this field and highlight the value of considering the influence of sex and gender in practice. These courses, such as “The Science of Sex and Gender in Human Health,” are designed to enable “researchers, clinicians and students in the health professions to integrate knowledge of sex and gender differences and similarities into their research and practice” [64].

### Is there momentum for change?

Longstanding efforts to improve the health of women have called for increasing the representation of women in clinical studies, analysis of research results by sex or gender, and awareness both within the research community and the public about the essential need to study the influence of sex and gender on health. Grassroots advocacy calling for change, new U.S. federal laws for the practice of research, the development of federally-funded studies on the health of women, and the establishment of interdisciplinary research collaborations and centers at academic health centers dedicated to women’s health have helped advance our understanding of the many health conditions that have sex and gender differences, or are unique to or more prevalent in women. In sum, the wide-ranging efforts of many people, groups and agencies have made this possible.

However, it is also clear from the history presented here that despite these hard won advancements, much more progress is still needed. Importantly, both increased public awareness and recent signs within key governmental agencies suggest that further progress may be within reach if we actively support the changes underway.

The National Institutes of Health, which is an influential leader in determining the direction of research, has begun to introduce new expectations for inclusion of female as well as male animals and sex-typing of cell lines in basic research [65]. Once these are approved by the White House Office of Management and Budget, the expectations will become requirements for applications submitted for the January 25, 2016 deadlines and beyond, and these will be the standard when reviewing grant proposals [66]. These would be new requirements because the changes implemented after the 1993 NIH Revitalization Act, requiring inclusion of women and minorities, excluded representation of females in laboratory studies of model systems and sex-typing cell lines that could not be traced back to a particular individual. As basic science research often lays the groundwork for much of the clinical trials that follow, the inclusion of females and cell typing by sex in laboratory studies could result in a momentous change in how we conduct science and what we will learn from scientific inquiry.

In addition, very recently, the U.S. Food and Drug Administration issued a proposed Action Plan to reduce barriers for participation and increase enrollment of women and minorities. Just as important, the Plan proposes to increase analysis of data by sex, age, race, and ethnicity, and to post data — including in the labeling of drugs and devices. Many, including members of the U.S. Congress from both sides of the aisle, recognize this FDA plan as a key opportunity to finally advance women’s health and reduce risk that is related to use of medications and medical devices.

Bolstering this new momentum, the U.S. Patient Protection and Affordable Care Act (ACA) codified the establishment of Offices on Women’s Health in major U.S. federal agencies, including Health and Human Services, the Centers for Disease Control and Prevention, and the Food and Drug Administration. While many of these offices existed before passage of the landmark law, the ACA prohibits their termination, reorganization, or transfers of powers and responsibilities without the approval of Congress [67].

Major print and television media outlets are also paying attention to the need for research on women’s health and gender differences (e.g. [50, 68]). As a result, the public has been made more aware of this issue and U.S. legislators and health officials have been prompted to call for new policies addressing sex and gender in health research.

Clearly, there is momentum now for progress. But how can we translate this movement into actions that advance research and improve health and health care? We propose that, first, it is necessary to examine and understand the primary barriers to inclusion and, second, to provide operational recommendations for change that surmount these barriers by including required actions on the part of all those participating in the conduct and translation of research.

### Barriers to full participation of women as research subjects and to the study of sex and gender differences

Until relatively recently, the prevailing scientific tradition was to not include women in clinical trials. Of note, even the first trial testing the effect of estrogen on secondary prevention of coronary heart disease, published in *The Journal of the American Medical Association* in 1973, was conducted solely with men, enrolling 8341 men and no women [69].

Three major reasons appear to account for the exclusion of women as research subjects, and even when women were included, not analyzing data by sex or gender.

First, women were ruled out of participation based on concerns about exposure to experimental risk during childbearing years. However, these concerns led to industry and government research policies that effectively excluded all women. Women’s health became conflated with women’s reproductive health and child health, and this narrow view persists in some quarters to this day. Furthermore, excluding women of childbearing age “assumes that women lack any control over their childbearing potential while participating in clinical trials” [70]. In fact, excluding all women of any age assumes that women, whether premenopausal or postmenopausal, cannot make informed judgments.

Protectionist perspectives have also represented women as being vulnerable to unwilling participation, and these perspectives still persist. For example, the Code of Federal Regulations of the NIH Office for Protection from Research Risks maintains criteria for Internal Review Board approval of research which includes the following provision: “When some or all of the subjects are likely to be vulnerable to coercion or undue influence, such as children, prisoners, pregnant women, mentally disabled persons, or economically or educationally disadvantaged persons, additional safeguards have been included in the study to protect the rights and welfare of these subjects” [71].

Over time, many overly protectionist policies that excluded women are coming to be viewed as discriminatory and lacking in scientific merit [16, 72]. However, some of these policies continue [71] and often remain in less obvious ways as well, for example through unconscious biases within the general culture [1, 73, 74], which results in women continuing to be underrepresented as participants in many areas of clinical research and in data on health outcomes infrequently analyzed by sex or gender. Clearly, the existence of unconscious bias must be recognized and tempered if we are to remedy its effects on sound scientific inquiry.

Second, misperceptions persist among some medical professionals and among segments of the public that women are less affected by certain disorders or health conditions and, when affected, that women respond to the same treatment as men. For example, cardiovascular disease (CVD), an area in which women have not been adequately studied, is the leading killer of women as well as men in the United States and most developed countries. Yet, as recently as 2005, an American Heart Association random survey of 500 physicians found that women were more likely than men to be deemed at lower risk of cardiovascular disease and less likely to be referred for diagnostic tests, despite similar risk. This study also found that “Fewer than 1 in 5 physicians knew that more women than men die each year from CVD” [75]. More recently, a 2007 study using a national sample of commercial health plans found that, despite similar access to care, women were less likely than men to receive recommended care for cardiovascular disease [76].

Cardiovascular disease also provides a clear illustration of treatment responses that can differ by gender. Studying only men, researchers learned in 1989 that low dose aspirin reduced the risk of a first heart attack in men 50 years and older by 44 % yet possibly increased stroke risk [28]. It took another 16 years for a study to show that low-dose aspirin was not effective in primary prevention of coronary artery disease risk in women under 65 but it was for women older than 65, and that aspirin reduced the risk of stroke in women 65 years and older [29].

The possibility of a sex-specific treatment response still is not being routinely taken into account. As shown in a 2010 survey of cardiovascular disease prevention clinical trials, published in *Circulation: Cardiovascular Quality and Outcomes*, “differences in treatment effect by sex were not commonly presented in the literature and that this practice has not changed over time” [3]. And, as demonstrated in the current report, cardiovascular disease is just one of many areas of health in which greater awareness and understanding of gender effects will improve health care.

Third, women are perceived as bringing complexity and thus increased cost and the need for greater analytical capability to scientific design. Including women in research studies, as the Institute of Medicine notes in its 2001 report, “introduces additional variables (in the form of hormonal cycles) and decreases the homogeneity of the study population” [8]. Yet, as the Institute of Medicine report also concludes, “being male or female is an important basic human variable that should be considered when designing and analyzing studies in all areas and at all levels of biomedical and health-related research.” Until sex and gender differences are routinely investigated and the results are routinely reported “many opportunities to obtain a better understanding of the pathogenesis of disease and to advance human health will surely be missed” [8].

Interestingly, in a survey of 10 different biological fields of study using non-human mammals and then in a meta-analysis of neuroscience and biomedical research studies using mouse models, biological variability was not found to be significantly greater in females than males. As pointed out in these studies, this finding, coupled with the occurrence of sex differences at “all levels of biological organization” warrant inclusion of female animals to ensure good science [77, 78]. Moreover, a recent 2014 Comment in Nature co-authored by the Director and Deputy Director of the National Institutes of Health points to ignoring sex differences as among the factors contributing to the growing concern of reduced scientific reproducibility in pre-clinical research [1].

In order to overcome these barriers, it is essential to implement consensus requirements for change. As the chronology in this report indicates, recommendations for actions to ensure the inclusion of sex and gender in research studies have been made by national academies, scientific committees, and leading scientists that have yet to be carried out. Whether existing or new, the following recommendations are valuable guides for implementing practices that improve our research capabilities and should be adopted now.

### Recommendations for change

1. The leading national agencies in biomedical research and in the approval of drug/device interventions, specifically the NIH and the FDA within the United States, must take the lead in both enforcing existing requirements and extending research design and analysis requirements to ensure adequate inclusion of women and investigations that provide meaningful analysis by sex and gender [1, 7, 63]. These strategies for inclusion should serve as a model for all government agencies that fund or conduct research [39].

#### With regard to the National Institutes of Health:

2. The opportunity to study women’s health and the influence of sex and gender on health should be central to the goals of the NIH and a key feature of the newly developing NIH-wide Strategic Plan [79].
  2.1. Development of both funding mechanisms and methodologies for collecting larger sample sizes that facilitate the analysis of sex and gender differences should be included in this plan [39].
    2.2. As part of the Strategic Plan, each Institute should be required to provide data annually on its independent funding of research on women’s health, its plan for establishing how the influence of sex and gender are routinely examined in mainstream research, and its contribution to collaborative funding with the NIH Office of Research on Women’s Health.
    2.3. When making funding decisions, priority should be given to proposals that show methods and designs providing for inclusion and analysis by sex and gender. As the journal *Nature* points out in a 2010 editorial, “Funding agencies should demand that researchers justify sex inequities in grant proposals and, other factors being equal, should favor studies that are more equitable” [80].
    2.4. In laboratory research using cell lines, the effects of the sex of cells must be investigated and reported [6–8].

#### With regard to the NIH and the FDA:

3. Research applications to the NIH that study women across varying risk subgroups, ages, race/ethnicities, social and behavioral factors should also be favored for research funding because not all women are the same [39]. Information and analyses on subgroups should also be part of FDA new drug applications. As the FDA’s recently issued Action Plan notes, “Making sure that different demographic subgroups are sufficiently represented in clinical trials and enhancing the analyses and public availability of subgroup data will contribute to development of a sound knowledge base as we move toward a time when all stages of patient care — from prevention to diagnosis to treatment to follow up — are truly personalized” [63].
4. The effects of hormonal factors on research outcomes should be investigated and understood as a further important refinement of subgroup differences with regard to reproductive status, the menstrual cycle, oral contraceptive use, menopause, and hormone therapies [8, 16].
5. In studying drug effects, both the sex differences in the way a drug is metabolized (pharmacokinetics) and the clinical response to drug concentrations (pharmacodynamics) should be investigated and reported. The empirical data that served as the rationale for this recommendation over 20 years ago in the *1993 FDA Guideline for the Study and Evaluation of Gender Differences in the Clinical Evaluation of Drugs* [16] continues to be replicated [24, 25, 81] and the FDA should be responsive to these findings in its requirements.
6. In studying medical devices, there must also be sex-specific data on safety and efficacy on all products [45, 47].
7. Reproductive considerations for participation in clinical intervention trials, such as for drug therapies, should be extended to men. If there is concern that exposure to an experimental condition could induce harm in reproductive capacity in women, parallel concern about inducing harm in men should be considered [70].

#### With regard to scientific publications and peer reviews:

8. Publishers and editors of all scientific and medical journals must expand existing policies and go beyond simply recommending inclusion of descriptive data on sex of subjects [82].
  8.1. Policies should ensure the inclusion and analysis of sex-specific data in clinical studies, as well as sufficient sample sizes to allow analysis of results by gender and sex. As the Institute of Medicine recommended in its 2010 report, “The International Committee of Medical Journal Editors and other editors of relevant journals should adopt a guideline that all papers providing the outcomes of clinical trials report on men and women separately unless a trial is of a sex-specific condition (such as endometrial or prostatic cancer)” [39]. The *Journal of the National Cancer Institute* was the first journal to include instructions to authors for addressing the effects of sex as part of its manuscript-preparation policy. And in fact, the practice and experience of this journal demonstrate that sex-specific analysis and reporting is possible, and “there has been no pushback since the institution of the policy” [5].
  8.2. The inclusion of female animals and cells in laboratory studies on health conditions suffered by women as well as analysis by sex should also be a prerequisite for manuscript consideration.
  8.3. It should be the responsibility of peer reviewers of both manuscripts and grant proposals to inquire as to these types of analyses if they are not provided. And as proposed by the Directors of the NIH and the ORWH, reviewers within the NIH review process should be “enjoined to evaluate applicants’ research plans to include, compare and contrast experimental findings in male and female animals and cells” [7].
  8.4. The Institute of Medicine’s 2012 workshop summary report provides a blueprint on sex-specific reporting strategies that can be adopted.
    8.4.1. For example, in reporting on clinical studies, the IOM report recommends: a) “The title and abstract should indicate whether a study involved only men or only women.” b) “If the study design allows identification of sex differences, journals should require authors to present these results.” c) “If there is an inability to identify sex differences, this should be reported in the discussions of the limitations of the study” [5].
    8.4.2. In reporting on preclinical studies, the IOM report recommends: a) “The sex of the animals studied should be reported.” b) “If only one sex of an animal was studied, this should be indicated in the title of the article.” c) “In most cases, the sex of origin of cells used should be reported (excluding, for example, immortalized cell lines, which are highly transformed and for which the sex of the original cells may not be relevant).” d) “Both male and female animals should be studied when appropriate; and, when it is possible, both sexes should be studied in the same experiment” [5].

#### With regard to training of scientists and clinicians:

9. The influence of sex and gender on health and treatment outcomes should be integrated into the training of researchers and clinicians [64].
  9.1. The scientific training of all clinical and laboratory biomedical researchers should focus on the value of including sex and gender as primary variables of study in order to enhance the precision and reproducibility of scientific inquiry. Learning how sex and gender affect outcomes can provide a basis for the motivation to embrace research that focuses on women’s health and sex differences.
  9.2. Findings derived from research on the influence of sex and gender on health must be incorporated into the education of health care professionals. Strategies for incorporating data on sex and gender health effects into health curricula are rapidly developing and professional resources are available to facilitate this process [83, 84].
  9.3. Similarly, medical educators must provide instruction in sex and gender effects on health in continuing medical education in order to ultimately optimize health care for all.

#### With regard to the translation and communication of research findings:

10. Sex-specific research findings should be provided to the community. After due diligence in ensuring the reliability and validity of scientific results, there must be a concerted effort by those overseeing the funding, direction and conduct of research along with clinical practice partners to ensure appropriate translation of findings and expedite this information for the benefit of all.
  10.1. The challenges in translating research results must be met by government agencies requiring researchers to discuss the translation of findings, and by professional medical associations requiring that clinical practice guidelines routinely incorporate sex-specific findings [39].
  10.2. Communication methods for new health information must be developed by academic health centers, federal agencies such as the NIH and FDA, and professional medical societies. Data of practical benefit must be disseminated in understandable language accounting for subgroup differences and possible conflicting data. Distribution of information through central clearinghouses, such as research institutes and professional society websites, should be coordinated and regulated to allow the public access to how research works and how to know what to take from its findings.

With regard to research, the Institute of Medicine’s 2012 report notes that “a culture shift within science” must occur to make the changes necessary to fully incorporate the study of sex and gender in health research [5]. We maintain that to effect a culture change it is necessary to turn the recommendations suggested here into requirements for research, coupled with rewards for following mandated guidelines. The approach to making positive change must be to reward and reinforce those who take a stand for gender-specific medicine, knowing that the change we make will result in advancements in well-being for both women and men.

Other actions also must be taken to advance the health of women and men. As healthcare consumers, we must ask our health care professionals about available research on how sex and gender may influence treatment decisions. Consumers and health care professionals alike need to express their support for research that has adequate representation of females and for analyses and reporting of study results by sex and gender. Federal and some state tax dollars provide much of the support for biomedical research in the United States, and legislators set the priorities and oversee the budgets for such government-supported research. Consequently, our lawmakers must be reminded of the importance of studying the effects of sex and gender across health conditions when making research funding decisions [85].

## Conclusion

Various efforts over the last 20 years have been made to ensure that the influence of sex and gender on health is part of our national research agenda and that research findings are translated into practice. As a function of continuing public scrutiny and congressional review over two decades, progress has been made. For example, the National Institutes of Health, which influences the direction of research, now requires that women be considered as subjects in NIH federally-funded clinical trials as prescribed by law and is expected to require the inclusion of females in studies using animal model systems and of sex-typing in studies of cells. The U.S. Food and Drug Administration, which regulates all prescription drugs and devices, has now issued an Action Plan which presumably will incorporate to some degree the study of sex differences in the approval process for drugs and devices. Due to the emergence of research illustrating the value of studying women and sex differences, public and professional awareness has grown as to how this is important to good science and to the public health.

However, progress has been slow and halting, and we have not achieved sufficient progress in order to truly serve the public health. To remedy this, specific recommendations for change are provided. For example, the NIH should offer priority to grant applications addressing sex and gender differences, and the FDA should require sex-specific data for all drug and device applications. The Institute of Medicine’s 2012 report notes that “a culture shift within science” must occur to make the changes necessary to fully incorporate the study of sex and gender in health research [5]. We maintain that to effect a culture change it is necessary to overcome the barriers to positive change by turning the recommendations suggested here into requirements for research, coupled with rewards for following mandated guidelines.

In addition, we must all participate in creating change, and accountability has to be built into the process of implementing change. This includes requiring that medical and continuing medical education incorporates consideration of the influence of sex and gender on health. The editors of medical journals should not consider studies that fail to include analysis of sex and gender unless studies are investigating conditions that affect one sex or gender. Consumers and legislators must also show their support for research that has appropriate representation of females and analyzes and reports results by sex and gender. With these types of changes and a commitment from all sectors, productive change is feasible and will herald better health for all.

## Abbreviations

AAOS: American Academy of Orthopaedic Surgeons
ACA: U.S. Patient Protection and Affordable Care Act
BIRCWH: Building Interdisciplinary Research Careers in Women’s Health
CVD: Cardiovascular disease
FDA: Food and Drug Administration
GAO: General Accounting Office, called the Government Accountability Office since 2004
IOM: Institute of Medicine
NIH: National Institutes of Heath
ORWH: Office of Research on Women’s Health
SCOR: Specialized Center of Research
WHI: Women’s Health Initiative
WHRY: Women’s Health Research at Yale
